# Conformation-stabilizing ELISA and cell-based assays reveal patient subgroups targeting three different epitopes of AGO1 antibodies

**DOI:** 10.3389/fimmu.2022.972161

**Published:** 2022-10-20

**Authors:** Christian P. Moritz, Le-Duy Do, Yannick Tholance, Pierre-Baptiste Vallayer, Véronique Rogemond, Bastien Joubert, Karine Ferraud, Coralie La Marca, Jean-Philippe Camdessanché, Jérôme Honnorat, Jean-Christophe Antoine

**Affiliations:** ^1^ Department of Neurology, University hospital of Saint-Etienne, Saint-Etienne, France; ^2^ Synaptopathies and autoantibodies (SynatAc) team, Mechanisms In Integrated Life Sciences (MELIS) Laboratory, Institute NeuroMyoGène (INMG), INSERM U1314/CNRS UMR 5284, Universités de Lyon, Université Claude Bernard Lyon 1, Lyon, France; ^3^ INMG/Melys team, University Jean Monnet, Saint-Étienne, France; ^4^ French Reference Center on Paraneoplastic Neurological Syndromes and Autoimmune Encephalitis, Hospices Civils de Lyon, Bron, France; ^5^ Department of Biochemistry, University Hospital of Saint-Etienne, Saint-Etienne, France; ^6^ European Reference Network for Rare Neuromuscular Diseases, Paris, France

**Keywords:** argonaute antibodies, sensory neuronopathy, ganglionopathie, Anti-Su antibodies, autoimmune neuropathy, conformation-stabilizing ELISA

## Abstract

Autoantibodies (Abs) are biomarkers for many disease conditions and are increasingly used to facilitate diagnosis and treatment decisions. To guarantee high sensitivity and specificity, the choice of their detection method is crucial. *Via* cell-based assays, we recently found 21 patients with neurological diseases positive for antibodies against argonaute (AGO), 10 of which having a neuropathy (NP). Here, we established a simple and conformation-sensitive ELISA with the aim to distinguish between AGO1 Abs against conformational epitopes and non-conformational epitopes and to reveal further characteristics of AGO1 antibodies in NP and autoimmune disease (AID). In a retrospective multicenter case/control and observational study, we tested 434 patients with NP, 274 disease controls with AID, and 116 healthy controls (HC) for AGO1 Abs *via* conformation-stabilizing ELISA. Seropositive patients were also tested for conformation-specificity *via* comparative denaturing/stabilizing ELISA (CODES-ELISA), CBA positivity, AGO1 titers and IgG subclasses, and AGO2 reactivity. These parameters were statistically compared among different epitope-specific patient groups. We found Abs in 44 patients, including 28/434 (6.5%) NP, 16/274 (5.8%) AID, and 0/116 (0%) HC. Serum reactivity was consistently higher for AGO1 than AGO2. Globally among the 44 AGO1 Abs-positive patients, 42 were also tested in CBA for AGO1 Abs positivity and 15 (35.7%) were positive. Furthermore, 43 were tested for conformation-specificity and 32 (74.4%) bound a conformational epitope. Among the subgroups of highly positive patients (ELISA z-score >14) with sera binding conformational epitopes (n=23), 14 patient sera were also CBA positive and 9 bound a second conformational but CBA-inaccessible epitope. A third, non-conformational epitope was bound by 11/43 (15.6%). Among the epitope-specific patient subgroups, we found significant differences regarding the Abs titers, IgG subclass, and AGO2 reactivity. When comparing AGO1 Abs-positive NP versus AID patients, we found the conformation-specific and CBA inaccessible epitope significantly more frequently in AID patients. We conclude that 1) conformational ELISA was more sensitive than CBA in detecting AGO1 Abs, 2) serum reactivity is higher for AGO1 than for AGO2 at least for NP patients, 3) AGO1 Abs might be a marker-of-interest in 6.5% of NP patients, 4) distinguishing epitopes might help finding different patient subgroups.

## Introduction

Autoantibodies (Abs) are biomarkers for many disease conditions and sometimes define entire specific entities. They are more and more used to facilitate diagnosis and treatment decisions (1, 2). Therefore, the choice of their detection method is crucial (3).

In the recent years, cell-based assay (CBA) has developed as a method of choice for antibody detection in neurological disease (4). An advantage of CBA is its ability to identify conformational epitopes that have greater chance to be involved in the disease pathogenesis. Disadvantages are difficulties to standardize the method due to variations in transfection success and subjectivity of data read-out, as well as a limited sensitivity, especially of standardized commercial approaches that may hence lose patients eligible to treatment (4).

For enzyme-linked immunosorbent assays (ELISA) in contrast, the conformation of target antigens in ELISA is less clear and commonly considered farther away from the native conformational state (5), while there are advantages in its use for high-throughput approaches. Hence, the ability of other methods than CBA to detect conformational epitopes is an object of concern, especially given that not all autoantigen epitopes are conformational. In detail, several antibodies recognize linear epitopes such as the anti-Hu (6) or anti-Yo (7) antibodies in paraneoplastic neurological disorders and they are nevertheless crucial for the identification of the associated disease.

We recently used CBA for the detection of anti-argonaute (AGO) Abs in neurological diseases. Twenty-one patients reacting with AGO1 and 2 were identified who had different neurological syndromes among which limbic encephalitis and sensory neuronopathy were the most frequent (8). AGO2 Abs have been reported in the serum of patients with systemic lupus erythematosus (SLE), scleroderma, Sjögren syndrome (SjS), and other rheumatologic autoimmune diseases (9). In our previous study, 67% of AGO Abs-positive patients had an associated systemic autoimmune disease and importantly, 62% improved with immunomodulatory treatments showing the potential importance of detecting AGO antibodies in certain neurological diseases (8). We concluded that AGO Abs might be potential biomarkers of an autoimmunity context in patients with central and peripheral neurologic disease, which might help predicting the treatment response.

The following questions have remained open and are aimed to be addressed in this study:

1) Is AGO1 or AGO2 the main target? While AGO2 has been described as the targeted antigen in autoimmune disease (AID)-related studies (9), we found both AGO1 and AGO2 to be targeted. No quantitative method has compared them yet.

2) Is the targeted epitope conformational or linear? There have been hints of a conformational epitope in some index patients in our first study (8) without being systematically addressed.

3) What is the proportion of anti-AGO Abs in patient cohorts with neuropathies (NP)?

4) What is the most appropriate method to detect AGO Abs? An ELISA approach might be preferable in terms of standardization and screening of large cohorts, but so far, only an ELISA with limited sensitivity and specificity has been described (10). It is unclear if ELISA approaches would be sensitive and specific enough to complement the CBA approach and to detect conformational epitopes.

5) Is there more than one epitope that are potentially able to distinguish biologically different subgroups?

## Material and methods

### Standard protocol approvals, registrations, and patient consents

The study was approved by the ethical committee of the University Hospital of Saint-Etienne (IRBN742021/CHUSTE), France, and was carried out in accordance with the Code of Ethics of the World Medical Association (Declaration of Helsinki). All participants provided written informed consent. The privacy rights of human subjects were observed. No animal experiments were conducted.

### Study design, patients, and controls

In the first part of the study, we established a comparative denaturing/stabilizing ELISA with a reduced sample size including 19 of our patients recently reported AGO1/AGO2 Abs-positive and 16 HC.

In the second part of the study, we chose the stabilizing version of ELISA to screen the entire series of patients and controls: 434 patients with NP including 132 patients with sensory neuronopathy, 116 with chronic inflammatory demyelinating polyneuropathy (CIDP), 81 with small fiber neuropathies (SFN), and 105 with axonal length dependent sensorimotor neuropathies of unknown origin. Sera were tested in a retrospective multicentric case/control and observational study. Controls consisted of 274 patients with AID and no peripheral neuropathies including 87 SLE, 146 SjS, and 41 autoimmune hepatitis, primary biliary cirrhosis, systemic sclerosis, vascularitis, myositis, rheumatoid arthritis, or juvenile arthritis (positive control cohort), and finally, 116 healthy controls (HC; negative control cohort).

### Serum collection

Sera from patients were obtained from our biobank (CRB42 CHU Saint-Etienne, France, AC 2018-3372, NFS96-900, N° of collection DC-2010-1108). HC sera came from the blood donation service of the French Blood Establishment. Samples were collected from October 1998 to January 2021, prepared and stored as previously (11, 12).

### Establishment of a comparative denaturing/stabilizing ELISA for anti-AGO1 Abs detection

We coated 1 µg/mL AGO1 or AGO2 protein in three coating conditions:

(1) standard coating buffer (0.05 M carbonate/bicarbonate, pH 9.6 (Sigma-Aldrich, Saint-Louis, USA),

(2) in-house-adapted conformation-stabilizing coating buffer (30% glycerol in coating buffer), and

(3) in-house-adapted denaturizing coating buffer (0.8% SDS during the 5-minute denaturation step with 100 µg/mL AGO1 stock solution, diluted to 0.008% SDS while dilution AGO1 to 1 µg/mL working solution), in order to linearize AGO1. The percentages of SDS were chosen in a way to be above the critical micelle concentration during the denaturation step but below it during the coating period, in order to avoid hindrance of the coating process.

For each serum, the serum-specific background noise (SSBN) was quantified by using uncoated but otherwise equally treated wells in parallel to each coated well (12). The SSBN was subtracted from each serum reactivity to obtain the specific binding (i.e., difference of optical densities of coated versus uncoated wells; delta optical density, ΔOD). After saturation with 3% BSA in PBS, sera were incubated at a 1/100 dilution overnight at 4 °C. The commercial rabbit-anti-AGO1 antibody (NBP1-56530, Novus Biologicals, Bio-Techne, Wiesbaden, Germany) was used in a 1:500 dilution. Secondary antibodies (1:3,000 for anti-human IgG, 1:1000 for anti-human IgG1, 1:200 for anti-human IgG2, 1:400 for anti-human IgG3, 1:700 for anti-human IgG4, same product references as recently (13) were applied for 2 h at 4 °C. Washing steps (2, 6, and 10 cycles after coating, serum incubation, and secondary antibody incubation, respectively) were done with 0.1% Tween-20 in PBS. ODs were systematically read after 30 minutes incubation with the substrate o-phenylenediamine dihydrochloride.

For each of the three coating conditions a respective ΔOD positivity threshold was defined as the arithmetic mean plus 3 standard deviations of the ΔODs of the 16 HC under the respective conditions.

To define the conformational status for each seropositive sample, we compared the results of the denatured and stabilized antigen, hence we called it comparative denaturing/stabilizing ELISA (CODES-ELISA). Patients with conformational AGO1-Abs epitopes were defined as those among AGO1 Abs-positive patients that lose ≥50% of their ELISA reactivity under denaturing as compared to stabilizing conditions. Patients with non-conformational epitopes were defined as those who lose <50% or even gain reactivity under denaturing as compared to stabilizing conditions.

### ELISA for AGO1 vs. AGO2 comparison, screening of the different disease cohorts, and IgG subclass identification

For 1) comparing AGO1 vs. AGO2 signals in the fourteen patients recently reported AGO1/AGO2 Abs-positive in CBA (8), 2) screening the larger disease cohorts, and 3) IgG subclass detection, we applied the conformation-stabilizing coating version (with glycerol) and our previously reported ELISA design (13, 14) using AGO1 as the antigen and the above described ELISA protocol.

“AGO Abs-positive” (ELISA+ or ELISA++) has been defined as a reactivity of ≥4 standard deviations (SD) above the mean reactivity of 116 (for AGO1 Abs) or 13 (for AGO2 Abs) healthy controls. “Moderately positive” (ELISA+) and “strongly AGO Abs-positive” (ELISA++) have been defined as a reactivity of 4-14 SD and ≥14 SD, respectively, above these mean reactivities of healthy controls.

The antibody titer was determined by serial dilutions of the sera in ELISA at 1/100, 1/1,000, 1/10,000, 1/100,000, and 1/1,000,000.

### Cell-based assays

All patients positive in ELISA were also tested in CBA using the same protocol as in our previous study (8). Briefly, HEK293 cells were transfected with VP5-HA-AGO1 or VP5-HA-AGO2, fixed, permeabilized, and incubated with 1:100 serum. Binding was revealed by using 1:200 Alexa555 fluorochrome-conjugated secondary goat anti-human IgG antibodies and a fluorescent microscope Axio Imager Z1. For each CBA, we carried out a double staining between an anti-HA antibody and anti-human IgG in order to verify the co-localization and thus avoid false positives.

### Comparison of patient groups regarding potentially differing epitopes

We compared the characteristics of Abs in three groups of AGO1 Abs-positive patients whose CBA and ELISA results suggested the potential existence of three different epitopes or groups of epitopes: 1) **epitope subgroup 1** with potential conformational epitope: patients with an ELISA++, conformation-specific in ELISA and CBA-positive; 2) **epitope subgroup 2** with potential conformational but CBA-non-accessible epitope: patients with an ELISA ++, conformation-specific in ELISA, and CBA-negative; 3) **epitope subgroup 3** with a non-conformational epitope: patients positive but non-conformation-specific in ELISA and CBA-negative.

To compare CBA-positive and CBA-negative patients (epitope group 1 and 2), we chose only patients being both strongly ELISA-positive (ELISA++) and conformation-specific with ELISA. This was done to avoid including patients that are CBA-negative solely due to a lower titer (but positive and conformation-specific with ELISA).

### Statistics

Categorical data were analyzed by the χ² test or by Fisher’s exact test and continuous data by the Kruskal-Wallis test, Mann-Whitney test or Student’s t-test depending on their distribution (Kolmogorov-Smirnov test). Quantitative results were expressed as median (interquartile range (IQR)) or mean ± standard error of mean (SEM) and categorical data were presented as count (percentage). A p-value of ≤0.05 was used to determine statistical significance. Statistical analysis was performed with MedCalc Statistical Software version 18.2.1 (MedCalc Software, Ostend, Belgium).

## Results

### Determining AGO1 as the main antibody target

**Figure 1:**
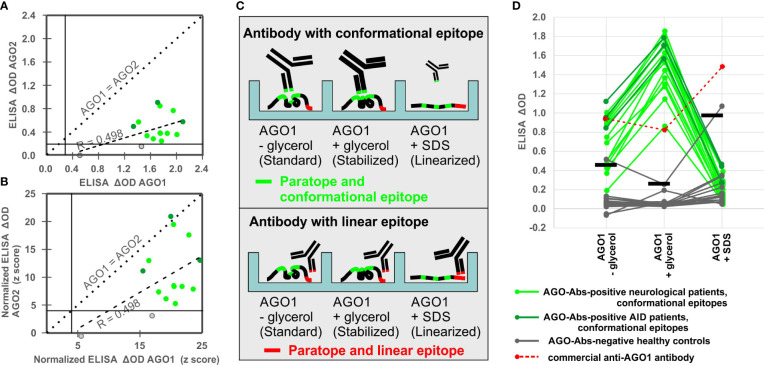
Comparison of AGO1 and AGO2 Abs and establishment of a denaturation assay to assess conformation-specificity of AGO1 Abs. **(A)** Correlation analysis between ELISA ΔODs for AGO2 and AGO1 Abs. **(B)** Correlation analysis between normalized ELISA ΔODs (z-scores) for AGO2 and AGO1 Abs. **(C)** Scheme of CODES-ELISA to distinguish antibodies with conformational and linear epitopes. Size and line width of schematic antibodies represent their binding reactivity. **(D)** ELISA denaturation assay using sera from 19 AGO Abs-positive patients, 16 healthy controls, and 1 commercial anti-AGO1 antibody. For each of the three conditions a respective positivity threshold was defined based on the ODs of the 16 healthy controls under the respective conditions (horizontal bars = arithmetic mean plus 3 standard deviations).

Fourteen of our patients recently reported AGO1/AGO2 Abs-positive in CBA (8) were tested for both AGO1 and AGO2 reactivity in ELISA in order to choose the more reactive antigen for this study. AGO1 and AGO2 reactivities (ΔODs) correlated moderately (r = 0.498; p = 0.055), but the AGO2 reactivities were consistently lower than the AGO1 reactivities throughout all sera (**Figure 1A**). While all 14 sera (100%) could be confirmed to be AGO1 Abs-positive in ELISA, only 12/14 (85.7%) were confirmed AGO2 Abs-positive. Even after normalizing the signals for each antigen with a respective control serum cohort of HC resulting in z-scores (i.e., the number of standard deviations above the average), AGO2 reactivities were in average still lower than AGO1 reactivities (**Figure 1B**). As these findings confirmed our recent results in protein microarrays (8), we concluded that AGO1 is the more sensitive antigen in ELISA and continued our method establishment with that antigen.

### Establishment of CODES-ELISA to assess conformation-specificity

Nineteen of our patients recently reported AGO1/AGO2 Abs-positive (8) and 16 HC were tested in our newly established CODES-ELISA, applying AGO1 protein in three conditions: standard coating (without glycerol), conformation-stabilizing coating (with glycerol), and denaturing coating (with SDS) in order to linearize AGO1 (**Figure 1C**).

In order to (1) confirm that the coating step has not been disturbed by the denaturation process and to (2) have a control anti-AGO1 antibody raised against a linear peptide, we have used a commercial anti-AGO1 antibody and found strong signal in all three conditions (red points and dashed lines in **Figure 1D**). The strongest signal was seen with the linearized AGO1, which was expected due to the linear epitope of commercial AGO1 Abs and thereby confirms the success of the linearization process **(Figures 1C, D**).

In contrast, all 19 AGO1 Abs-positive patients showed the opposite pattern, i.e., they considerably lost ELISA reactivity with the non-stabilized or linearized AGO1 (green points and lines in **Figure 1D**). None of the 19 sera (0%) was positive under denaturizing conditions, while 1/16 of the HC turned false-positive (6.3%). ELISA under standard conditions (without glycerol) confirmed only 14/19 CBA-positive sera (73.7%), while 1/10 of the HC was false-positive (10%).

Only under the conformation-stabilizing conditions (with glycerol) all 19 (100%) AGO 1Abs-positive sera were confirmed *via* ELISA, while at the same time none (0%) of the 16 HC was positive (**Figure 1D**). Regarding the signal intensity, using conformation-stabilizing conditions significantly increased the reactivity of the AGO1 Abs-positive sera by 112.2% (from ΔOD = 0.70 to ΔOD = 1.50; p<0.0001). Denaturing the antigen led to a significant loss of 85.5% of the reactivity compared to conformation-stabilizing conditions (from ΔOD = 1.50 to ΔOD = 0.22; p<0.0001). We concluded that conformation-stabilizing conditions should be used for AGO1 Abs detection in order to have the highest sensitivity and specificity of antibody detection *via* ELISA. Hence this procedure was then used to screen a larger population of patients and controls in the following experiments.

Furthermore, by comparing AGO1 in three different coating conditions, we did not only find a sensitive and specific ELISA approach. At the same time this CODES-ELISA design represented a tool to distinguish AGO1 Abs with conformational epitopes, i.e., those that significantly lose their reactivity upon antigen linearization, from AGO1 Abs with rather linear epitopes, i.e., those that do not lose enough or even gain reactivity upon antigen linearization. Hence, this denaturation assay was later used to assess the conformation-specificity of all identified AGO1 Abs-positive subjects.

### Frequency of the AGO1 Abs in NP and AID

**Figure 2:**
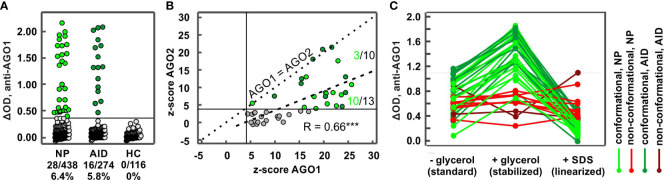
Frequencies in different disease groups and characteristics of AGO1 Abs. **(A)** ELISA ΔODs plotted for different disease groups comprising 824 subjects. Each data point represents one subject. Each green data point (44 in total) above the horizontal line at ΔOD = 0.386 (i.e., z-score = 4) represents a AGO1 Abs-positive subject. NP: neuropathies, AID: autoimmune disease, HC: healthy controls. **(B)** Correlation analysis between normalized ELISA ΔODs (z-scores) for AGO2 and AGO1 Abs. Dotted line: z-score AGO1 = z-score AGO2; Dashed line: linear correlation curve. Numbers above and below the dashed curve represent the proportion of AGO1/AGO2 Abs-positive NP sera. *** p ≤ 0.001. **(C)** ELISA denaturation assay using sera from 43 AGO Abs-positive patients (27 with NP and 16 with AID). Green curves: patients with conformation-specific reactivity pattern; red curves: patients with non-conformation-specific reactivity pattern.

We applied our established conformation-stabilized ELISA approach to detect AGO1 Abs-positive subjects in a cohort of 824 sera of patients with NP, AID, and healthy blood donors. AGO1 Abs were found in 44 patients, including 28/434 (6.5%) NP, 16/274 (5.8%) AID, and 0/116 (0%) HC (**Figure 2A**). Regarding the different disease groups among the neuropathies, 17 of 132 (12.9%) SNN patients, 4 of 116 (3.6%) CIDP patients, 3 of 81 (3.7%) SFN patients, and 4 of 105 (4.0%) ONP were AGO1-Abs-positive. While the Abs frequency in both PN and AID was significantly higher than in the healthy controls (NP: p = 0.005; AID: p = 0.008), there were no significant differences between NP and AID (p = 0.74).

### Characteristics of AGO1 Abs including conformation specificity

We found a significant and better correlation between the normalized AGO1 and AGO2 Abs ELISA reactivities than with the smaller cohort from our first experiment (r = 0.66; p < 0.001; cf. **Figure 1A**) and the sensitivity for AGO1 Abs was in average still higher than for AGO2 Abs (**Figure 2B**). Nevertheless, four AGO Abs-positive patient sera had an equal AGO1 and AGO2 reactivity. Three of those were AID and only one NP patient. Furthermore, among patients positive for both AGO1 and AGO2 Abs, NP patients were more likely to lie below the correlation curve than above it (10/13 vs. 3/10, p = 0.04), confirming that NP patients are more reactive for AGO1 than for AGO2 and suggesting that the ratio of AGO1/AGO2 reactivity is higher in NP than in AID patients. AGO1 Abs titers correlated significantly with AGO1 Abs ELISA reactivity (r = 0.74; p <0.001; not shown).

**Figure 3:**
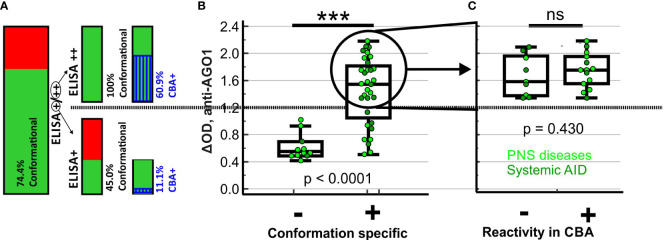
Proportions, ELISA reactivities and disease characteristics of sera with conformational or CBA-positive AGO1 Abs. **(A)** Bar charts representing proportion of subjects with conformation-specific AGO1 Abs among all AGO1-ELISA-positive subjects, on the right side split up in strongly (++) and moderately (+) ELISA-positive subjects together with another bar chart showing the proportion of AGO1-CBA-positive subjects among those with conformation-specific AGO1 Abs. **(B)** ELISA-reactivity of all AGO1-ELISA-positive subjects plotted depending on conformation specificity. For moderately positive (+) subjects, colors of single spots represent disease group. **(C)** ELISA-reactivity of all strongly (++) AGO1-ELISA-positive subjects with conformation-specific AGO1 Abs plotted depending on CBA reactivity. Colors of single spots represent the disease group of the corresponding subject. *** p < 0.001; ns = not significant; Kruskal-Wallis test.

Among the 44 AGO Abs-positive patients, 43 were tested for conformation-specificity and 32 (74.4%) bound a conformational epitope (**Figure 2C**, **Figure 3A**), while the other 11/43 (15.6%) bound a non-conformational epitope. Regarding reactivities, for all patients with a conformational epitope, conformation-stabilizing conditions significantly increased the reactivities of the AGO1 Abs-positive sera by 90% (from ΔOD = 0.74 to ΔOD = 1.41; p<0.0001). Denaturing the antigen led to a significant loss of 83.7% of the reactivity compared to conformation-stabilizing conditions (from ΔOD = 1.41 to ΔOD = 0.23; p<0.0001). In contrast, for the patients with non-conformational epitopes, conformation-stabilizing conditions did not change the reactivities significantly (2.2% increase from ΔOD = 0.59 to ΔOD = 0.60; p = 0.86), while antigen denaturation likewise did not change the reactivity significantly compared to conformation-stabilizing conditions (8% loss from ΔOD = 0.60 to ΔOD = 0.55; p = 0.28).

The proportion of subjects with conformational antibodies was significantly higher among the ELISA++ samples (23/23, 100%) than among the ELISA+ samples (9/20, 45.0%; p < 0.001; cf. **Figure 3A**). Likewise, among all AGO1-positive subjects, the ELISA reactivities of subjects with conformational AGO1 Abs was significantly higher than that of subjects without (**Figure 3B**, p ≤ 0.001). Among the 23 subjects with AGO1 Abs that are both conformational and ELISA++, 14 (60.9%) were CBA-positive. Thus, there are still 9 (39.1%) that are CBA-negative without showing any reduced ELISA reactivity (**Figure 3C**, p = 0.43). This finding may refer to a second epitope that – although conformational – is accessible in ELISA but not in CBA. Among the 9 tested subjects with AGO1 Abs that are conformational and moderately (+) ELISA-positive, 1 (11.1%) was CBA-positive. The presence of another patient group of 11/43 (25.6%) subjects with AGO1 Abs epitopes that are non-conformational may represent of a third epitope. All these subjects were CBA-negative. Globally among the 44 AGO1 Abs-positive patients, 42 were tested in CBA and 15 (35.7%) were positive by this method (data not shown). We conclude that ELISA was more sensitive in detecting AGO1 Abs than CBA.

### Comparison of disease repartition, antibody titers, and IgG subclasses between potential epitope-depending subgroups

We see evidence for three epitope candidates enabling the separation of three non-intersecting subgroups of AGO1 Abs-positive patients: 1) patients with antibodies targeting epitope-1 (conformational and CBA-accessible), 2) patients with antibodies targeting epitope-2 (conformational but CBA-inaccessible), 3) patients with antibodies targeting epitope-3 (non-conformational).

We compared these three subgroups regarding a set of parameters and found significant differences regarding the AGO1 Abs titers, IgG subclass distribution, and AGO2 Abs-positivity (chi^2^ among the three groups, p < 0.001, p < 0.001 – 0.05, p < 0.001, respectively, **Table 1**). In detail, high titer AGO1 Abs were more frequent in the epitope-1 and -2 subgroups versus epitope-3 subgroup. Moreover, IgG1 was more frequent in epitope-1 versus epitope-3 subgroups, and IgG4 was more frequent in epitope-1 versus epitope-2 and -3 subgroups. More than one single IgG subclass was detected less frequently in epitope-3 compared to epitopes-1 and -2 subgroups. Furthermore, the epitope-3 subgroup was less frequently AGO2 Abs-positive than the other two subgroups (**Table 1**). Regarding the disease group repartitioning, we only found a trend towards more AID and less NP in the epitope-2 compared to the epitope-3 subgroup.

**Table 1:** Comparing the three epitope subgroups regarding disease repartition, titers, IgG subclasses, and AGO2 Abs positivity.

Category	Epitope 1 (conf./CBA+)	Epitope 2 (conf./CBA-)	Epitope 3 (non-conf.)	p-value (Chi^2^)
**Disease repartition**				
** NP**	10/14 (71.4%)	3/9 (33.3%)	9/11 (81.8%)	0.06
** SNN**	8/10 (80.0%)	3/3 (100%)	6/9 (66.7%)	0.47
** AID**	4/14 (28.6%)	6/9 (66.7%)	2/11 (18.2%)	0.06
**Titers**				
** >=10,000**	12/14 (85.7%)**^§^**	4/9 (44.4%)**^§^**	0/11 (0%)**^#,$^**	**0.0001**
** <10,000**	2/14 (14.3%)**^§^**	5/9 (55.6%)**^§^**	11/11 (100%)**^#,$^**	**0.0001**
**IgG subclass**				
** IgG1**	14/14 (100%)**^§^**	9/9 (100%)	7/11 (63.6%)**^#^**	**0.009**
** IgG2**	2/14 (14.3%)	0/9 (0%)	0/11 (0%)	0.22
** IgG3**	3/14 (21.4%)	4/9 (44.4%)**^§^**	0/11 (0%)**^$^**	**0.050**
** IgG4**	8/14 (57.1%)**^$,§^**	1/9 (11.1%)**^#,§^**	0/11 (0%)**^#^**	**0.003**
** >1 subclass**	11/14 (78.6%)**^§^**	5/9 (55.6%)**^§^**	0/11 (0%)**^#,$^**	**0.0001**
**Other features**				
** anti-AGO2-positive**	12/13 (92.3%)**^§^**	8/9 (88.9%)**^§^**	1/9 (9.1%)**^#^**	**0.0001**

Absolute numbers (percentage); bold p-values are significant (p ≤ 0.05). #: significantly different from epitope 1; $: significantly different from epitope 2; §: significantly different from epitope 3.

To test if the higher titers for the conformational epitopes-1 and -2 are barely caused by a selection bias (only ELISA++ used for these two epitopes), we performed a comparison between all conformational versus non-conformational epitopes including both ELISA+ and ELISA++ samples. The titers were still significantly higher for the conformational epitopes (data not shown).

In order to find potential differences between AGO1 Abs-positive NP and AID patients, we compared those two groups regarding the same parameters as above both in an analysis comprising all patients and another analysis performed per epitope group (**Table 2**).

**Table 2:** Comparing NP and AID regarding epitope parameters, titers, and IgG subclasses.

Parameters	Total			Epitope 1 (conf./CBA+)			Epitope 2 (conf./CBA-)			Epitope 3 (non-conf.)		
	NP	AID	p-value	NP	AID	p-value	NP	AID	p-value	NP	AID	p-value
**Epitopes**												
** 1 (conf./CBA+)**	10/22 (45.5%)	4/12 (33.3%)	0.72									
** 2 (conf./CBA-)**	3/22 (13.6%)	6/12 (50%)	**0.04**									
** 3 (non-conf)**	9/22 (40.9%)	2/12 (16.7%)	0.25									
**Titers**												
** >=10,000**	10/28 (35.7%)	6/16 (37.5%)	1.00	9/10 (90.0%)	3/4 (75.0%)	0.51	1/3 (33.3%)	3/6 (50.0%)	1.00	0/0 (0%)	0/2 (0%)	-
** <10,000**	18/10 (64.3%)	10/16 (62.5%)	1.00	18/10 (64.3%)	10/16 (62.5%)	0.51	2/3 (66.7%)	3/6 (50.0%)	1.00	9/9 (100%)	2/2 (100%)	–
**IgG subclass**												
** IgG1**	24/28 (85.7%)	15/16 (93.8%)	0.64	10/10 (100%)	4/4 (100%)	1.00	3/3 (100.0%)	6/6 (100%)	–	6/9 (66.7%)	1/2 (50.0%)	1.00
** IgG2**	1/28 (3.6%)	1/16 (6.3%)	1.00	1/10 (10.0%)	1/4 (25.0)	0.51	0/3 (0%)	0/6 (0%)	-	0/9 (0%)	0/2 (0%)	-
** IgG3**	6/28 (21.4%)	4/16 (25.0%)	1.00	2/10 (20.0%)	1/4 (25.0%)	1.00	2/3 (66.7%)	3/6 (50.0%)	1.00	0/9 (0%)	0/2 (0%)	–
** IgG4**	6/28 (21.4%)	6/16 (37.5%)	0.30	5/10 (50.0%)	3/4 (75.0%)	0.51	0/3 (0%)	1/6 (16.7%)	1.00	0/9 (0%)	0/2 (0%)	-
** >1 subclass**	11/28 (39.3%)	10/16 (62.5%)	0.21	7/10 (70%)	4/4 (100%)	0.51	2/3 (66.7%)	4/6 (66.7%)	1.00	0/9 (0%)	0/2 (0%)	–
**Epitope features**												
** Conformational**	18/27 (66.7%)	14/16 (87.5%)	0.17	10/10 (100%)	4/4 (100%)	1.00	3/3 (100.0%)	6/6 (100%)	–	0/9 (0%)	0/2 (0%)	–
** CBA-positive**	11/26 (42.3%)	4/16 (25.0%)	0.33	10/10 (100%)	4/4 (100%)	1.00	0/3 (0%)	0/6 (0%)	-	0/9 (0%)	0/2 (0%)	-
** anti-AGO2-positive**	12/27 (44.4%)	7/16 (43.8%)	1.00	8/10 (80.0%)	4/4 (100%)	1.00	3/3 (100.0%)	5/6 (83.3%)	1.00	1/9 (11.1%)	0/2 (0%)	1.00
** OD AGO1/AGO2 ratio**	5.6 (4.6 - 9.4)	5.3 (2.5 - 9.5)	0.32	4.6 (4.0 - 6.0)	2.4 (1.9 - 3.2)	**0.03**	8.1 (–)	5.3 (2.5 - 8.3)	0.38	9.7 (3.7 - 24.1)	49.9 (-)	0.22

For qualitative data: absolute numbers (percentage); for quantitative data: median (95% CI); bold p-values are significant (p ≤ 0.05).

We found two significant differences: first, regarding all AGO1 Abs-positive patients, those targeting epitope 2 were significantly more frequent in AID than in NP patients, supporting the hypothesis of a potential clinical relevance in taking the epitopes into account. Second, among all patients targeting epitope-1, the ΔOD AGO1 Abs/ΔOD AGO2 Abs ratio was significantly higher in NP than in AID, supporting our hypothesis of a differing AGO1/AGO2 reactivity in NP versus AID, at least for one of the epitopes (cf. **Figure 2B**).

## Discussion

Referring to the five open questions listed in the Introduction, in this study we demonstrated evidence that 1) AGO1 is the main target of the recently described AGO Abs in NP patients, 2) AGO1 epitope are mostly, but not aways, conformational, 3) anti-AGO1 Abs occurred in about 6% of NP and AID patient cohorts, 4) a conformation-specific ELISA approach is more sensitive than CBA, and 5) there are at least three epitopes being potentially able to distinguish biologically different subgroups.

AGO Abs have been first described (and called anti-Su Abs) in autoimmune diseases in the beginning of the 1980s (15) and AGO2 has been presented as the targeted antigen in 2006 (16). However, in the last decade, AGO Abs have disappeared into the “death-valley of autoantibodies” (17) that might play interesting roles but have become neglected “orphans” (17) of the research community. To investigate a potential clinical role of AGO Abs in larger cohorts, the research community needs simple and easily standardizable detection methods. As patient antibodies appear to predominantly bind the native AGO proteins, immunoprecipitation has initially been considered the only method to reliably detect anti-Su/Ago2 antibodies (9). Nevertheless, while we confirmed that denatured (Western blot) or truncated AGO proteins were not useful to detect these antibodies, our recently established CBA appeared reliable to detect the native antigen (8). Here, we established a sensitive ELISA approach which we applied to estimate the frequency of AGO1 in NP and AID and to determine sensitivity differences between AGO1 and AGO2 Abs.

We found similar frequencies of AGO1 Abs of about 6% in AID and NP. This frequency is in the range of what has been described for AGO2 Abs in different AID. In detail, AGO2 Abs have been described in 3-24% of SLE patients, 3-32% of scleroderma/systemic sclerosis patients, 0-9% of rheumatoid arthritis patients, and 8-13% of primary SjS patients (9), and 0-9% of polymyositis/dermatomyositis patients (10), but also in 5% of hepatitis B virus or hepatitis B+C virus coinfected patients (18), and 3% of breast cancer patients (19). Although we find a correlation between AGO1 and AGO2 Abs with a higher sensitivity for AGO1 Abs, we cannot be sure whether there is only one antibody partly reacting with both AGO proteins or whether there are two different antibodies. Pre-adsorption or immune-purification studies would be necessary to address this issue, however, as most of these methods are established for epitope peptides, prior knowledge about the exact binding domain would be helpful. While AGO2 Abs have been the antibody detected in AID during the last decades, we here find evidence for AGO1 Abs being more common in NP. No diagnostic value has been described for AGO2 Abs in AID. In contrast, we assume that AGO1 (and potentially also AGO2) Abs might be a biomarker for an autoimmune context in NP. We are currently performing a clinical comparison between AGO1 Abs-positive and -negative NP to address the question of a potential diagnostic value in neurology.

Here, by comparing AGO in three different conditions *via* CODES-ELISA, we established a sensitive and specific ELISA for the detection of AGO1 Abs. At the same time, the CODES-ELISA design represented a tool to distinguish AGO Abs targeting different epitopes. Antibodies with conformational epitopes lose more than 80% of their reactivity upon antigen linearization, which was the case for most of the AGO1 Abs-positive patient sera. Antibodies with rather non-conformational epitopes do not or not significantly lose their reactivity upon antigen linearization, such as the commercial anti-AGO1 antibody and a subgroup (here: subgroup-3) of AGO1 Abs-positive patients. Using this approach, all 14 tested patients from our first study bound a conformational epitope, which confirms our first suspicion, where denatured (Western blot) or truncated (one spot of the protein arrays) AGO proteins were not or less useful to detect these antibodies (8).

Although the conformation-stabilizing properties of glycerol are known for many years (20), its use in the coating buffer for ELISA is not common to our experience. In our study, the presence of glycerol doubled the reactivity of conformation-specific AGO1 Abs-positive sera. Therefore, we strongly recommend using our antigen-stabilizing protocol for AGO1 Abs analytics. Also for antigens other than AGO1, the usage of glycerol should be considered in order to bring the antigen closer to its native conformation. For example, MOG Abs are considered to only be specifically identified *via* CBA, while ELISA causes unspecific binding in some subjects (21). As we found a reduced specificity for AGO1 ELISA (1 out of 10 HC turned false-positive) when using classical or denaturizing conditions, we recommend testing the sensitivity and specificity for conformation-stabilized ELISA for anti-MOG, but also other antigens.

We see several advantages of conformation-stabilized ELISA compared to CBA. 1) In our study, ELISA was more sensitive than CBA, as expected from the study of other autoantigens (22), without losing specificity, since all the controls remained negative. As a rough rule, those patients that have the highest antibody titer in ELISA are positive in CBA. 2) Using only a single antigen in the wells and in combination with the correction of SSBN, the presence of other autoantibodies in the patient sera does not disturb the results. In CBA, in contrast, we considered those sera as false-positive, that were about equally reactive with the non-transfected cells. True-positive reactivity may be masked by false-positive reactivity in some cases. 3) In our hands, ELISA appeared to be more reproducible than CBA, although we did not quantitatively test this impression. 4) ELISA permits the detection of linear epitopes and CODES-ELISA permits the distinction of linear and conformational epitopes. In total, conformation-stabilized ELISA is probably the method of choice for anti-AGO1 Abs detection.

We conclude that most (about 75%) of the AGO1 Abs-positive patients bind a predominantly conformational epitope. However, a subgroup of those patients (about 20% of all seropositives) being strongly ELISA-positive but CBA-negative may bind another conformational epitope that was only detected by ELISA. A third group (about 25% of seropositives) is binding a rather linear epitope. Expectedly, these latter patients were CBA-negative.

Having more than one epitope is not uncommon. Among FGFR3 Abs-positive neuropathy patients for example, we recently found five different epitopes on the FGFR3 protein. These epitopes comprised even functional sites and clinical patterns were different among the patients targeting different epitopes (23).

For AGO1 Abs, we did not intend to reveal the exact epitope sequence in this study, as this is difficult to realize for conformational epitopes. The SPOT technology used for anti-FGFR3 Abs does not work in the case of anti-AGO1 Abs as the serum of most seropositive patient would not bind non-conformational peptides. Instead, mutated or otherwise transgenically modified conformational full-length proteins would be necessary to find the epitopes.

Nevertheless, we were able to elucidate some properties of the epitope candidates. The antibodies binding to conformational epitopes (epitopes-1 and -2) had higher titers than the non-conformational epitope-3. Moreover, IgG subclasses 1, 3, and 4 are less frequently detected in patients targeting non-conformational epitopes. IgG4 is in addition more frequent in patients targeting the CBA-accessible epitope-1 compared to those targeting CBA-inaccessible epitope-2. The conformation-specificity of IgG4 antibodies is in line with the special role of this IgG subclass enabling to block or even dimerize target proteins (24, 25), which requires conformal epitopes. The additional difference in its frequency between the two conformational epitopes might stress potential differences in functional roles of AGO1 Abs in different epitope-related patient subgroups. However, this difference is probably not linked with the disease group, as we did not find a significant difference of IgG4 antibodies frequency between NP and AID.

By choosing only ELISA++ and conformation-specific sera to defines the patient subgroups for epitope-1 and -2, we aim at excluding sera that are simply CBA-negative due to low antibody titers or non-conformational epitopes and we therefore assume to compare two truly different epitope groups: patients with a CBA-accessible epitope versus those with a CBA-non-accessible epitope. However, although we may have reduced the risk of a biased conclusion, we still cannot exclude that subgroup-2 is CBA-negative due to lower antibody titers than subgroups-1. Although there is no significant difference between the percentage of patients with high AGO1-Abs titers between the two subgroups, the percentage tends to be higher in subgroup-1. Regarding the disease repartitioning we found only trends toward a higher proportion of AID and a lower proportion of NP in patients targeting epitope-2 compared to those targeting epitope-3. However, when comparing different parameters between AID and NP, we found a higher proportion of epitope-2 targeting patients in AID. This potential preference of AID patients for the CBA-inaccessible epitope-2 might 1) explain why CBA detected much less AID than ELISA (apart from sensitivity differences) and 2) be a potential way to distinguish AGO1 Abs-positive AID from AGO1 Abs-positive NP patients.

As a last finding that stresses potential differences between epitopes, the AGO1/AGO2 Abs reactivity was significantly higher in NP versus AID in patients targeting CBA-accessible epitope-1. This is in line with our finding of more NP than AID patients being with a strong AGO1>AGO2 pattern in our correlation study (cf. **Figure 2B**). As a simplified conclusion, we see evidence for AGO2 Abs being more pronounced in AID and AGO1 Abs being more pronounced in NP, which needs to be validated with larger patient cohorts however.

It is not uncommon to find sensitivity differences between ELISA and CBA. Titers of neutralizing antibodies obtained by cell-culture-based neutralization assays for example do not correlate with antibody titers from ELISA for mumps and measles virus in human (22). For anti-GAD65 antibodies, a very similar sensitivity difference between ELISA and CBA has been reported as we found for anti-AGO1: While all 39 sera with high anti-GAD65 titers (>10,000) were CBA-positive, almost all sera (50/52) with lower anti-GAD65 ELISA titers (<10,000) were negative (26).

In contrast to anti-AGO1, only patients with high anti-GAD65 titers showed some improvement after immunotherapy. For neurofascin, contactin-1, and contactin-associated protein 1 autoantibodies in CIDP, even if all seropositive patients have been reported to be positive in both ELISA and CBA, the necessity of a less diluted CBA speaks for a higher sensitivity of ELISA (27).

To conclude, by establishing and applying conformation-stabilizing ELISA, we found similar frequencies of AGO1 Abs of about 6% in AID and NP. A more detailed analysis focusing disease sub-categories, including a clinical characterization of the antibody is ongoing (Moritz/Tholance et al., manuscript in preparation). This current study proposes a cheap and easily standardizable approach combining advantages of ELISA (better standardizable, better sensitivity and better adapted to high-throughput screenings) and advantages of CBA (conformation often closer to “real” native conformation) as a method of choice for AGO Abs detection. With this approach, at least 3 putative epitopes, conformational and non-conformational ones, with the ability to distinguish different patient subgroups occur with AGO1 Abs. By using different epitopes, a distinction between AGO1 and AGO2 Abs and/or between AID and NP might be possible. However, the necessity of conformational epitopes for most of the patients currently impedes the application of peptide assays. So far, the CODES-ELISA in parallel with CBA presented in this study appears as the only way of detecting the epitope-depending subgroups of AGO1 Abs-positive patients. As patients with anti-AGO1 Abs, even for low titer, might be prone to improve with immunomodulatory treatments (manuscript in preparation), due to its better sensitivity ELISA is to be recommended for AGO1 Abs detection in patient with PN.

## Data availability statement

The raw data supporting the conclusions of this article will be made available by the authors, without undue reservation.

## Ethics statement

The studies involving human participants were reviewed and approved by the Ethical committee of the University Hospital of Saint-Etienne (IRBN742021/CHUSTE), France. The patients/participants provided their written informed consent to participate in this study.

## Author contributions

CPM, YT, J-PC, and J-CA contributed to conception and design of the study. KF, CPM, and YT organized the database. CLM, CPM, L-DD, and VR performed the experiments. YT, P-BV and CPM performed the statistical analysis. CPM and J-CA wrote the first draft of the manuscript. YT wrote sections of the manuscript. All authors contributed to the article and approved the submitted version.

## Funding

This work has been developed within the BETPSY project, which is supported by a public grant overseen by the French National Research Agency (ANR), as part of the second “*Investissements d´Avenir*” program (reference ANR-18-RHUS-0012), and FRM (*Fondation pour la Recherche Médicale*) DQ20170336751. Parts of the study were funded by the Association Française contre les Myopathies (AFM-MyoNeurALP project 6.1.1) and the Fonds de dotation CSL Behring pour la recherche. The funders had no role in study design, data collection and analysis, decision to publish, or preparation of the manuscript. Work has been funded by the Centre Hospitalier Universitaire de Saint-Étienne.

## Acknowledgments

We thank Evelyne Federspiel, Chloé Pabiou, and Axel Maniak for assistance in ELISA. We thank all funding institutions listed in the appropriate chapter that supported our study. We appreciate the contribution of the reviewers who helped improving this manuscript. J-PC and J-CA are members of the European Reference Network for Rare Neuromuscular Diseases, 75013 Paris, France.

## Conflict of interest

CPM, L-DD, J-PC, JH, J-CA have submitted a patent application for the application of AGO antibodies as biomarkers for autoimmune neurological diseases.

The remaining authors declare that the research was conducted in the absence of any commercial or financial relationships that could be construed as a potential conflict of interest.

## Publisher’s note

All claims expressed in this article are solely those of the authors and do not necessarily represent those of their affiliated organizations, or those of the publisher, the editors and the reviewers. Any product that may be evaluated in this article, or claim that may be made by its manufacturer, is not guaranteed or endorsed by the publisher.

## References

[1] Jia Y Li M Li D Zhang M Wang H Jiao L. Immune-mediated cerebellar ataxia associated with neuronal surface antibodies. Front Immunol (2022) 13:813926. doi: 10.3389/FIMMU.2022.813926 35250990 PMC8891139

[2] Endmayr V Tunc C Ergin L de Rosa A Weng R Wagner L. Anti-neuronal IgG4 autoimmune diseases and IgG4-related diseases may not be part of the same spectrum: A comparative study. Front Immunol (2022) 12:785247. doi: 10.3389/FIMMU.2021.785247 35095860 PMC8795769

[3] Kharlamova N Dunn N Bedri SK Jerling S Almgren M Faustini F. False positive results in SARS-CoV-2 serological tests for samples from patients with chronic inflammatory diseases. Front Immunol (2021) 12:666114. doi: 10.3389/FIMMU.2021.666114 34012450 PMC8126683

[4] Woodhall M Mgbachi V Fox H Irani S Waters P. Utility of live cell-based assays for autoimmune neurology diagnostics. J Appl Lab Med (2022) 7:391–3. doi: 10.1093/JALM/JFAB133 PMC8741325 34996083

[5] Peery HE Day GS Dunn S Fritzler MJ Prüss H de Souza C. Anti-NMDA receptor encephalitis. the disorder, the diagnosis and the immunobiology. Autoimmun Rev (2012) 11:863–72. doi: 10.1016/J.AUTREV.2012.03.001 22440397

[6] Trier NH Hansen PR Vedeler CA Somnier FE Houen G. Identification of continuous epitopes of HuD antibodies related to paraneoplastic diseases/small cell lung cancer. J Neuroimmunol (2012) 243:25–33. doi: 10.1016/J.JNEUROIM.2011.12.020 22264992

[7] O’Donovan B Mandel-Brehm C Vazquez SE Liu J. Parent a v Et al High-resolution epitope mapping of anti-hu and anti-yo autoimmunity by programmable phage display. Brain Commun (2020) 2:fcaa106. doi: 10.1093/BRAINCOMMS/FCAA059 PMC7425417 32954318

[8] Do LD Moritz CP Muñiz-Castrillo S Pinto AL Tholance Y Brugiere S. Argonaute autoantibodies as biomarkers in autoimmune neurologic diseases. Neurology(R) Neuroimmunology Neuroinflamm (2021) 8:e1032. doi: 10.1212/NXI.0000000000001032 PMC8362341 34321331

[9] Satoh M Chan JYF Ceribelli A Del-Mercado MV Chan EKL. Autoantibodies to argonaute 2 (su antigen). Adv Exp Med Biol (2013) 768:45–59. doi: 10.1007/978-1-4614-5107-5_4 23224964

[10] Ogawa-Momohara M Muro Y Satoh M Akiyama M. Autoantibodies to Su/Argonaute 2 in Japanese patients with inflammatory myopathy. Clinica Chimica Acta (2017) 471:304–7. doi: 10.1016/j.cca.2017.06.022 28673815

[11] Moritz CP Stoevesandt O Tholance Y Camdessanché J-P Antoine J-C. Proper definition of the set of autoantibody-targeted antigens relies on appropriate reference group selection. New Biotechnol (2021) 60:168–72. doi: 10.1016/j.nbt.2020.08.007 33045420

[12] Moritz CP Tholance Y Rosier C Reynaud-Federspiel E Svahn J Camdessanché J-P. Completing the immunological fingerprint by refractory proteins: Autoantibody screening *via* an improved immunoblotting technique. Proteomics - Clin Appl (2019) 13:e1800157. doi: 10.1002/prca.201800157 30768763

[13] Tholance Y Moritz CP Rosier C Ferraud K Lassablière F Reynaud-Federspiel E. Clinical characterisation of sensory neuropathy with anti-FGFR3 autoantibodies. J Neurology Neurosurg Psychiatry (2020) 91:49–57. doi: 10.1136/jnnp-2019-321849 31690697

[14] Moritz CP Tholance Y Lassablière F Camdessanché J-P Antoine J-C. Reducing the risk of misdiagnosis of indirect ELISA by normalizing serum-specific background noise: The example of detecting anti-FGFR3 autoantibodies. J Immunol Methods (2019) 466:52–6. doi: 10.1016/j.jim.2019.01.004 30654043

[15] Treadwell EL Alspaugh MA Sharp GC. Characterization of a new antigen-antibody system (su) in patients with systemic lupus erythematosus. Arthritis Rheumatism (1984) 27:1263–71. doi: 10.1002/art.1780271108 6497921

[16] Jakymiw A Ikeda K Fritzler MJ Reeves WH Satoh M Chan EK. Autoimmune targeting of key components of RNA interference. Arthritis Res Ther (2006) 8:R87. doi: 10.1186/AR1959 16684366 PMC1779426

[17] Fritzler MJ Choi MY Satoh M Mahler M. Autoantibody discovery, assay development and adoption: Death valley, the Sea of survival and beyond. Front Immunol (2021) 12:679613. doi: 10.3389/fimmu.2021.679613 34122443 PMC8191456

[18] Vázquez-Del Mercado M Sánchez-Orozco LV Pauley BA Chan JYF Chan EKL Panduro A. Autoantibodies to a miRNA-binding protein Argonaute2 (Su antigen) in patients with hepatitis c virus infection. Clin Exp Rheumatol (2010) 28:842–8. 21122261

[19] Vázquez-Del Mercado M Martínez-García EA Daneri-Navarro A Gómez-Bañuelos E Martín-Márquez BT Pizano-Martínez O. Presence of anti-TIF-1γ, anti-Ro52, anti-SSA/Ro60 and anti-Su/Ago2 antibodies in breast cancer: A cross-sectional study. Immunopharmacol Immunotoxicology (2021) 43:328–33. doi: 10.1080/08923973.2021.1910833 33876712

[20] Gekko K Timasheff SN. Mechanism of protein stabilization by glycerol: preferential hydration in glycerol-water mixtures. Biochemistry (1981) 20:4667–76. doi: 10.1021/BI00519A023 7295639

[21] Reindl M di Pauli F Rostásy K Berger T. The spectrum of MOG autoantibody-associated demyelinating diseases. Nat Rev Neurol (2013) 9:455–61. doi: 10.1038/nrneurol.2013.118 23797245

[22] Brgles M Kurtović T Lang Balija M Hećimović A Mušlin T Halassy B. Impact of complement and difference of cell-based assay and ELISA in determination of neutralization capacity against mumps and measles virus. J Immunol Methods (2021) 490:112957. doi: 10.1016/J.JIM.2021.112957 33412172

[23] Tholance Y Antoine J-C Mohr L Jung M Reynaud-Federspiel E Ferraud K. Anti-FGFR3 antibody epitopes are functional sites and correlate with the neuropathy pattern. J Neuroimmunol (2021) 361:577757. doi: 10.1016/J.JNEUROIM.2021.577757 34768040

[24] Koneczny I. A new classification system for IgG4 autoantibodies. Front Immunol (2018) 9:97. doi: 10.3389/FIMMU.2018.00097 29483905 PMC5816565

[25] Cao M Koneczny I Vincent A. Myasthenia gravis with antibodies against muscle specific kinase: An update on clinical features, pathophysiology and treatment. Front Mol Neurosci (2020) 13:159. doi: 10.3389/FNMOL.2020.00159 32982689 PMC7492727

[26] Muñoz-Lopetegi A de Bruijn MAAM Boukhrissi S Bastiaansen AEM Nagtzaam MMP Hulsenboom ESP. Neurologic syndromes related to anti-GAD65: Clinical and serologic response to treatment. Neurology(R) Neuroimmunology Neuroinflamm (2020) 7:e696. doi: 10.1212/NXI.0000000000000696 PMC7136051 32123047

[27] Cortese A Lombardi R Briani C Callegari I Benedetti L Manganelli F. Antibodies to neurofascin, contactin-1, and contactin-associated protein 1 in CIDP: Clinical relevance of IgG isotype. Neurology(R) Neuroimmunology Neuroinflamm (2019) 7:e639. doi: 10.1212/NXI.0000000000000639 PMC6935837 31753915

